# Comparison of Different Suture Techniques on Nasal Soft Tissue Envelope to Prevent Supratip Deformity: A Histologic Study

**DOI:** 10.1007/s00266-024-04041-x

**Published:** 2024-04-18

**Authors:** Serhat Şibar, Ayhan Işık Erdal, Duygu Şibar, Süheyla Esra Özkoçer

**Affiliations:** 1 Department of Plastic, Reconstructive and Aesthetic Surgery, Faculty of Medicine, Gazi University, 06500 Ankara, Turkey; 2 Department of Histology and Embryology, Faculty of Medicine, Gazi University, 06500 Ankara, Turkey

**Keywords:** Rabbits, Supratip deformity, Scarring, Rhinoplasty, Sutures

## Abstract

**Background:**

Studies have described various surgical maneuvers in rhinoplasty to limit thickening from excessive scarring in the supratip region. This study aimed to compare the effectiveness of three maneuvers—a simple suture, a U suture, and bolster dressing—used to avoid supratip deformity in a rabbit model.

**Methods:**

Thirty-two male New Zealand white rabbits were included. The animals were divided into four groups, and dissection was performed in the supra-perichondrial plane up to the supratip region through an open rhinoplasty incision. After dissection, the following approaches were applied to the supratip region: Group 1, simple approximation suture; Group 2, U suture; Group 3, bolster dressing; and Group 4 (control group), no suture. All animals were sacrificed after 12 weeks. Histological analysis was performed.

**Results:**

In Group 4, scar thickness was significantly greater than in the other groups (*p* < 0.05). Group 3 had greater scar thickness than Group 2 (*p* < 0.05). The ratio of scar thickness to skin thickness was higher in Group 4 compared with the other groups (*p* < 0.05). Finally, there was no difference in the ratio of scar thickness to skin thickness between Groups 1, 2, and 3 (*p* > 0.05).

**Conclusions:**

In this study, it was concluded that surgical methods using sutures in the supratip region reduced scar thickness in a rabbit model, and these surgical methods had similar levels of effectiveness.

**No Level Assigned:**

This journal requires that authors assign a level of evidence to each submission to which Evidence-Based Medicine rankings are applicable. This excludes Review Articles, Book Reviews, and manuscripts that concern Basic Science, Animal Studies, Cadaver Studies, and Experimental Studies. For a full description of these Evidence-Based Medicine ratings, please refer to the Table of Contents or the online Instructions to Authors www.springer.com/00266.

## Introduction

Supratip deformity is one of the main problems seen after rhinoplasty. In addition to creating suboptimal aesthetic and functional results, this deformity can adversely affect patients’ personal and professional lives and self-confidence. For these reasons, secondary (revision) surgeries may be inevitable. Theoretically, the transition from the dorsum to the tip is observed in an aesthetically ideal nose. The supratip region is slightly behind the tip in the profile view (supratip break).

In contrast, supratip deformity occurs when convexity or increased thickness develops in the supratip area after surgery. In cases with supratip deformity, the supratip region is more anterior than the tip in the profile view. In the literature, incidences ranging from 3.6 to 56 percent have been reported, representing a wide range [1–3].

Etiologically, supratip deformity can be divided into two scenarios, depending on whether it originates from cartilage or soft tissue [4]. While the treatment of cartilage-based supratip deformity is the excision of excess cartilage, the physiopathology and treatment of soft tissue-based supratip deformity are more complex. The reasons for this are the dead space that occurs after excision and unpredictable scar tissue formation. In recent years, with a better understanding of the importance of soft tissue and skin envelope on healing, obliterating the dead space in the supratip region has become essential for achieving predictable healing [5–7]. Placing sutures in the supratip area is a frequently used method for this purpose, and different suture techniques have been described. The supratip approximation suture, defined by Guyuron [2], is a simple suture in which the subcutaneous tissue in this area is fixed to the underlying cartilage. Hoehne et al. [8] modified this suture, fixing the subcutaneous tissue in the supratip region to the medial crura; they reported that the tension in the supratip region could be adjusted in this way. In another study, Tosun et al. [9] used a version of Hoehne’s technique that was suitable for experimental study on rabbits. Moreover, Küçükgüven et al. [10] described a technique in which dead space is eliminated with external bolster sutures in the supratip region. The senior author (SS) used the suture mentioned above techniques in his rhinoplasty practice when needed. Although all these techniques are reported to be clinically effective, comparing them histologically has not been possible. This study aimed to compare the histological changes caused by these three different suture techniques in the supratip region.

## Methods

Thirty-two young adult male New Zealand white rabbits weighing 2.5–3 kg were used in the study. The study was approved by the Animal Experiments Local Ethics Committee (G.U.ET-22.104). The subjects were divided into four groups, with eight rabbits in each group.

### Surgical Procedures

**Fig. 1:**
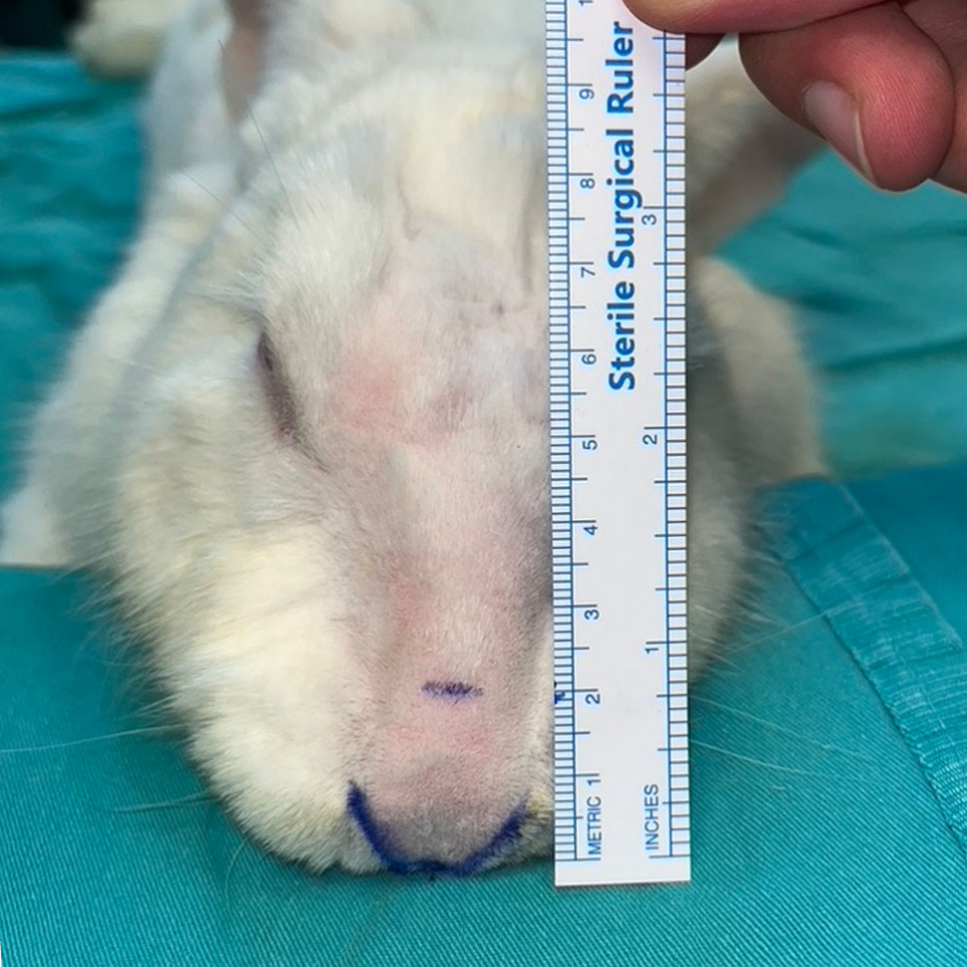
Determination of the standard suture placement zone in the supratip region

Anesthesia was induced with an intramuscular injection of ketamine (45 mg/kg) and xylazine (5 mg/kg). To minimize possible variables when applying the technique, all procedures were performed by the same surgeon (SS). After disinfection with a povidone-iodine solution, the nasal soft tissue envelope was elevated through an open rhinoplasty incision (transcolumellar and bilateral marginal). Dissection was continued through the areolar tissue in the supra-perichondrial plane until the upper and lower lateral cartilages and the caudal part of the nasal bones were seen. In all study groups, the suture was applied to the zone located 2 cm cranial to the tip of the nose (Fig. 1). The suture material was 5-0 polydioxanone on round needles.

**Fig. 2:**
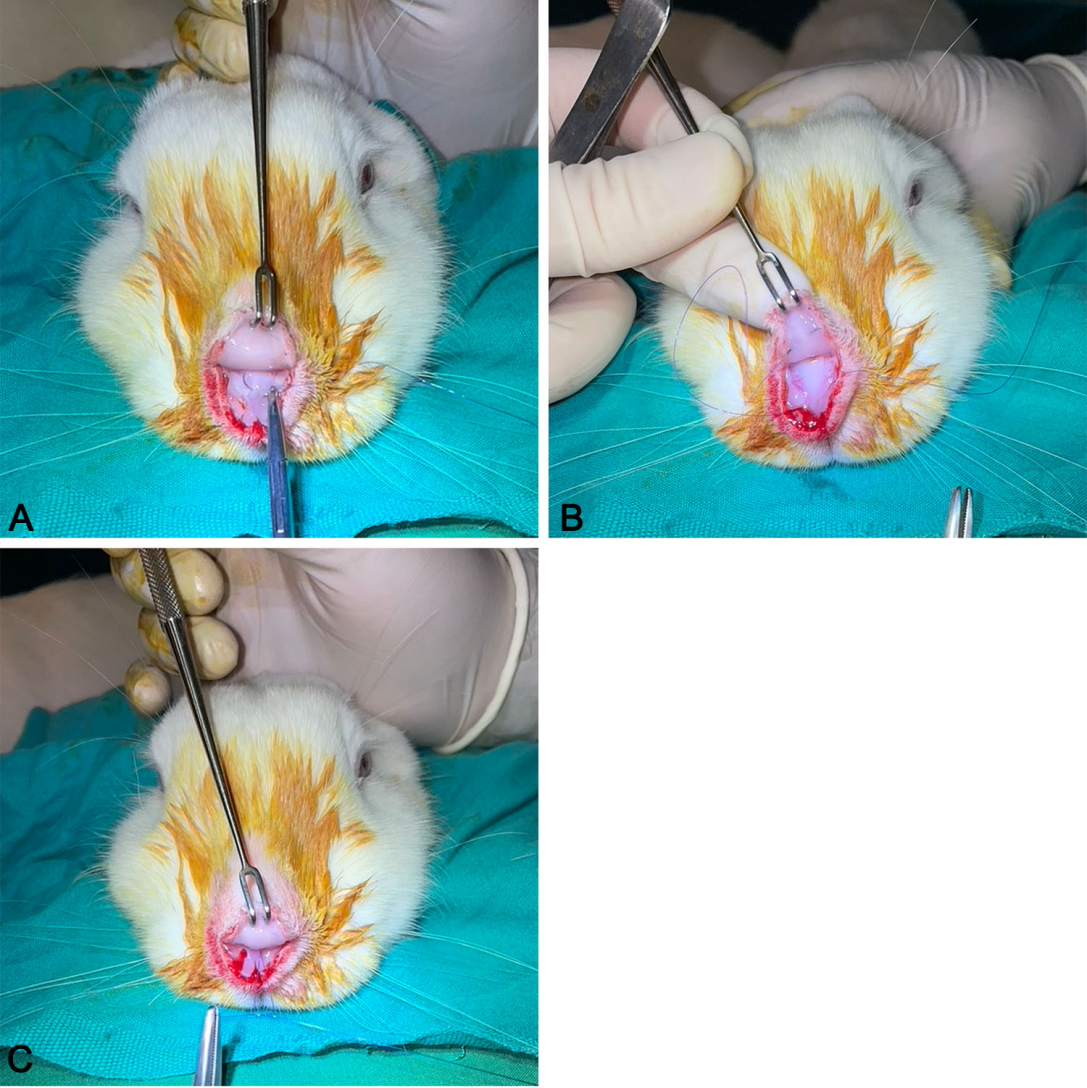
Placement of a simple approximation suture in Group 1. **A** The suture is passed dorsal to the dorsal septum. **B** The suture is passed dorsal to the nasal soft tissue envelope. **C** The simple suture is knotted

**Fig. 3:**
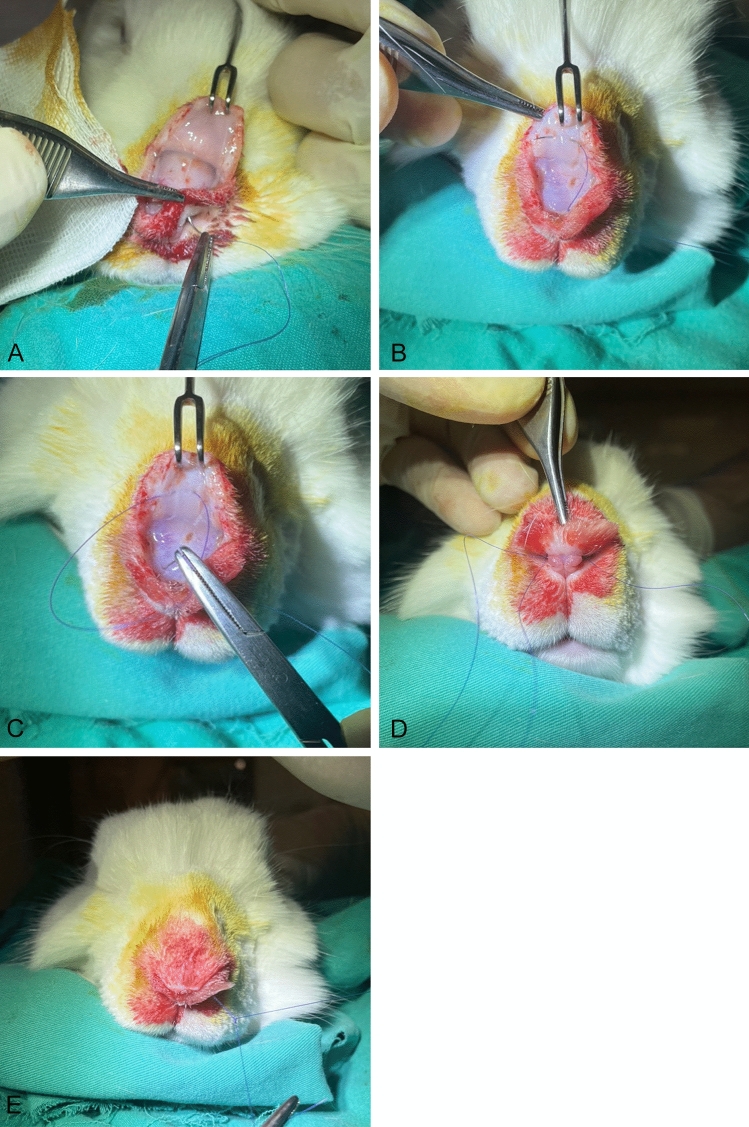
Placement of a U suture in Group 2. **A** The suture is passed from the left nasal cavity to the supratip region. **B** The suture is passed dorsal to the nasal soft tissue envelope. **C** The suture is passed to the right nasal cavity. **D** The suture is passed from the right nasal cavity to the left nasal cavity. **E** The U suture is knotted

**Fig. 4:**
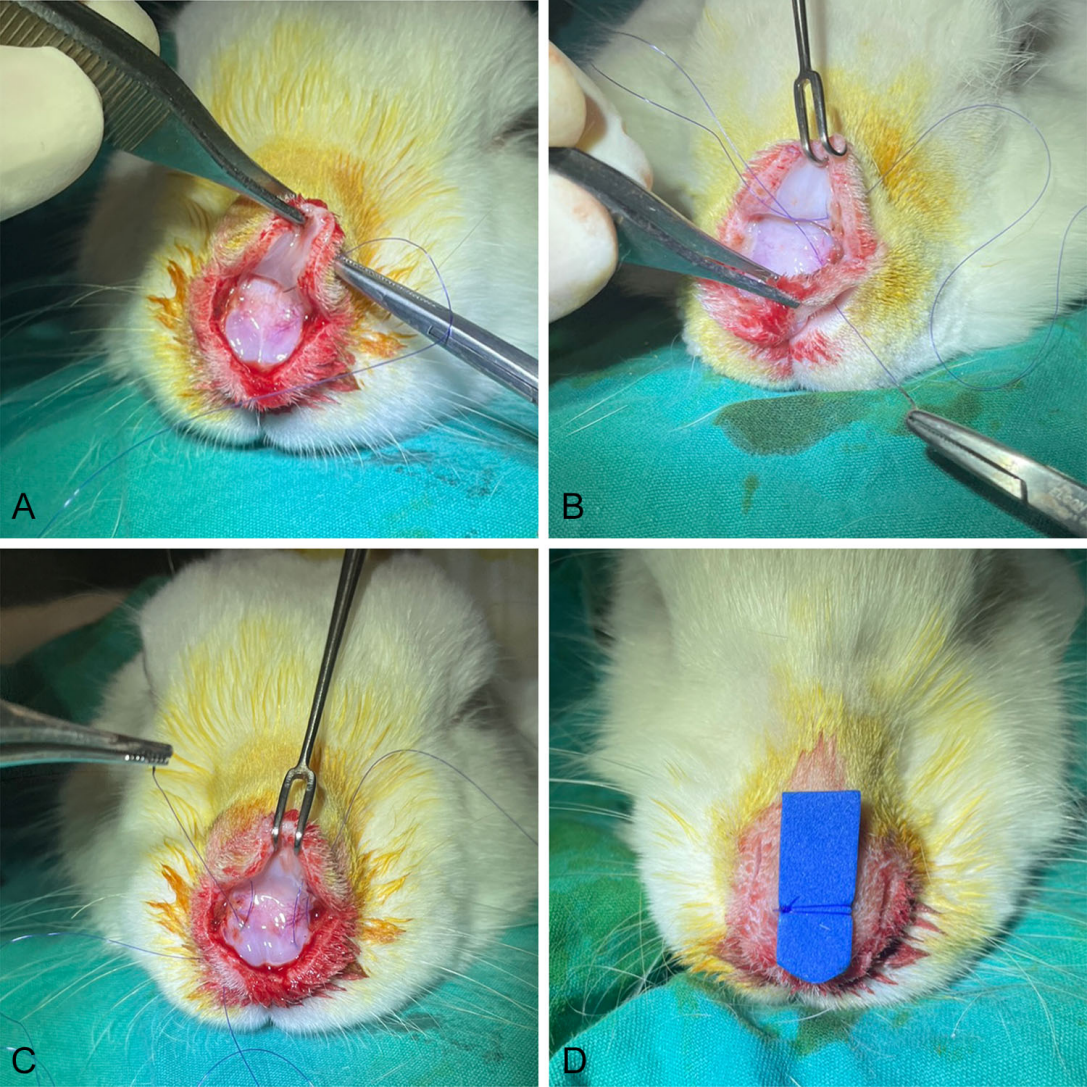
Placement of bolster dressing in Group 3. **A** The suture is passed from the nasal skin to the supratip region. **B** The suture is passed to the left nasal cavity. **C** Loops are created after multiple passes. **D** The suture is knotted over a sponge as a bolster dressing

In Group 1 (simple suture group), the superficial musculoaponeurotic system (SMAS) in the supratip region was fixed to the dorsal septum using the technique described by Guyuron et al. [2] (Figure 2). In Group 2 (U suture group), the technique defined by Tosun et al. [9] was applied. In the U suture technique, suture passes are started from the internal nasal valve region in the left nasal cavity; the suture is passed through the supratip area, SMAS tissue, and right nasal cavity, reaching the left nasal cavity via a transseptal route. The suture was then knotted in the left nasal cavity, bringing the supratip region closer to the septum (Fig. 3). In Group 3 (bolster dressing group), an external bolster dressing similar to the one that Küçükgüven et al. [10] described was applied to the supratip area; this was done by placing a sponge measuring 5 mm wide and 20 mm long in the area and fixing it with sutures (Fig. 4). Sponges were removed on the 10th day after the procedure. In Group 4 (control group), no suture was applied to the supratip region after dissection.

All rabbits were euthanized after being monitored for 12 weeks after the surgical procedure. No circulatory disorders or necrosis were encountered in the skin due to the suture technique used in any subjects. A full-thickness biopsy of skin, subcutaneous tissue, and cartilage was taken from the previously determined standard treatment zone in the supratip region (2 cm cranial to the nose tip), and histological analysis was performed.

### Histological Analysis

**Fig. 5:**
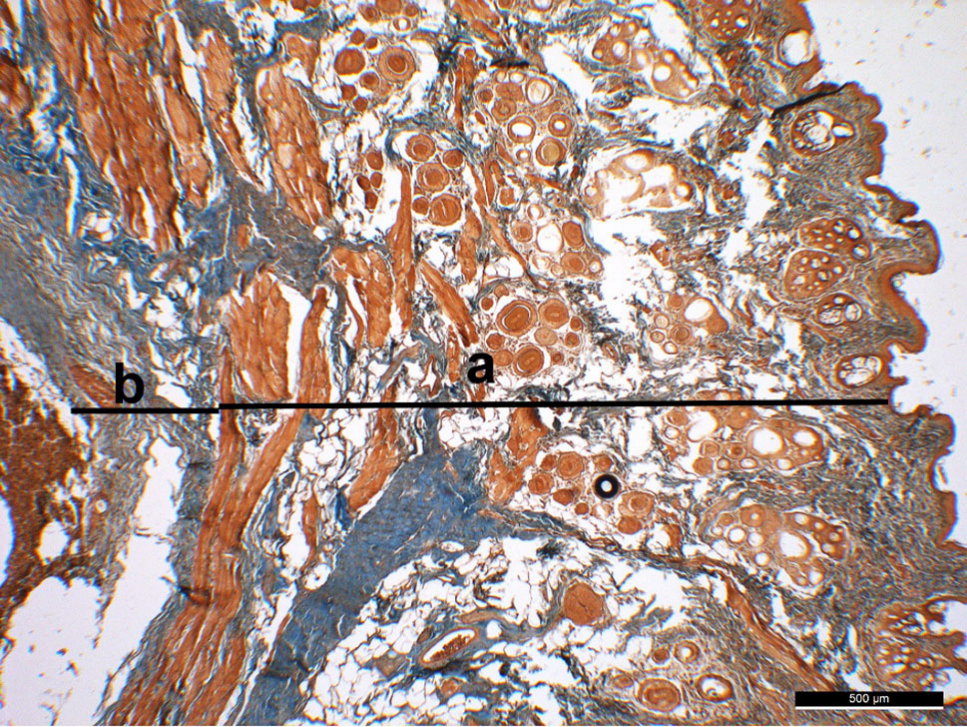
Measurement made on a tissue section stained with Masson’s trichrome stain. The thickness of the skin **a** and the thickness of the subcutaneous scar tissue **b** in the supratip area are shown

Specimens were fixed in 10% neutral buffered formalin. After processing, the tissues were embedded in paraffin. Sections of 4 µm in thickness were taken from formalin-fixed paraffin-embedded tissue specimens. Hematoxylin-eosin and Masson’s trichrome staining were used to evaluate the scar tissue thickness, epidermis, dermis, hypodermis, subcutaneous muscle layer, and newly formed subcutaneous collagen and the scar thickness [9]. The two following measurements were taken by an expert histologist: (a) the distance from the epidermis to the subcutaneous muscle layer and (b) the distance from the subcutaneous muscle layer to the lower border of the subcutaneous collagen layer. Measurements were repeated on six random sites for each section. The obtained values were analyzed statistically (Fig. 5).

### Statistical Analysis

SPSS Statistics (v.29.0; SPSS Inc., Chicago, IL) was used for the statistical analysis. Descriptive statistics were given using the mean and standard deviation because the quantitative data were normally distributed. A one-way analysis of variance (ANOVA) test was used to compare more than two independent groups, and the homogeneity of variances was evaluated with Levene’s test. If the variances were not homogeneous, Welch’s ANOVA test was used. In cases where there was a significant difference between groups, Tukey’s test was used to make pairwise post hoc comparisons if the variances were homogeneous. In contrast, Tamhane’s test was used if they were not homogeneous. The type 1 margin of error (alpha) was accepted as 0.05 for all statistics.

## Results

All the rabbits survived until the end of the experiment. No complications were observed. The average skin thickness in the supratip region was 2.36 mm (±0.28 mm) in Group 1, 2.24 mm (±0.18 mm) in Group 2, 2.26 mm (±0.35 mm) in Group 3, and 2.38 mm (±0.18 mm) in Group 4. The groups had no significant difference regarding average skin thickness (*p* >  0.05).

The scar tissue thickness results for the supratip region were 0.51 mm (±0.17 mm) in Group 1, 0.38 mm (±0.09 mm) in Group 2, 0.51 mm (±0.05 mm) in Group 3, and 0.85 mm (±0.07 mm) in Group 4. Significant differences in scar thickness were found between the groups (*p* < 0.05). Specifically, significant differences were detected in pairwise comparisons between Groups 1 and 4, Groups 2 and 4, Groups 3 and 4, and Groups 2 and 3 (*p* < 0.05).

The ratios of scar tissue thickness to skin thickness in the supratip region were 0.22 in Group 1, 0.17 in Group 2, 0.23 in Group 3, and 0.35 in Group 4. There were significant differences between the groups regarding these ratios (*p* < 0.05). Specifically, significant differences were detected in pairwise comparisons between Groups 1 and 4, Groups 2 and 4, and Groups 3 and 4 (*p* < 0.05) (Table 1).

**Table 1:** Intergroup comparative analysis of the skin thickness, scar tissue thickness, and ratio of scar tissue thickness to skin thickness in the supratip region

Groups	Supratip skin thickness (mm) (±SD)	Supratip scar thickness (mm) (±SD)	Ratio of scar thickness to skin thickness
Group 1 (Simple suture)	2.36 (±0.28)	0.51 (±0.17)^a^	0.22^e^
Group 2 (U suture)	2.24 (±0.18)	0.38 (±0.09)^b,d^	0.17^f^
Group 3 (Bolster dressing)	2.26 (±0.35)	0.51 (±0.05)^c,d^	0.23^g^
Group 4 (Control)	2.38 (±0.18)	0.85 (±0.07)^a,b,c^	0.35^e,f,g^
*p*-value	0.022*	<0.001^+^	<0.001^+^

*: ANOVA test, ^+^: Welch’s test,

There were statistically significant differences between Groups 1 and 4^a^, between Groups 2 and 4^b^, between Groups 3 and 4^c^, between Groups 2 and 3^d^, between Groups 1 and 4^e^, between Groups 2 and 4^f^, and between Groups 3 and 4^g^ in intergroup comparisons

## Discussion

In addition to shaping the bone and cartilage structures in rhinoplasty, it is crucial to control the healing of the nasal soft tissue envelope to achieve optimal results in the long term. Healing of the soft tissue envelope may vary depending on many factors, such as the surgical technique used, skin thickness, age, accompanying comorbid conditions, and lifestyle.

The postoperative healing response of each case varies individually. It is known that edema and inflammatory processes are more common in the postoperative period, especially in individuals with a thick, soft tissue envelope [11–13]. In addition, the formation of dead space between the soft tissue envelope and the bone and cartilage framework triggers fibrosis in the long term, causing changes in the soft tissue envelope. These changes are most evident in the supratip region, making predictability difficult. Guyuron reported that to prevent this problem, dead space should be eliminated with simple approximation sutures [2]. The suture techniques we used in our study (simple suture, U suture) and bolster dressing are methods reported in the literature to ensure control over the soft tissue envelope in the supratip region [2, 9, 10]. In a similar experimental model to the present study, Tosun et al. compared groups with sutures placed in the supratip region, external taping in the supratip area, and control groups. It was reported that the scar thickness in the supratip area was significantly less in the suture-placed group compared with the other two groups [9]. In our study, the methodology of the study by Tosun et al. was developed by evaluating the comparative effectiveness of simple suture, U suture, and bolster dressing on the same experimental model. At the same time, an experimental model of other suture techniques used in this region was defined.

The significant difference in scar thickness in the supratip area between the control and other groups showed that the treatments applied to the supratip area effectively reduced scar thickness. Since the scar thickness in Group 2 was thinner than that of Group 3, it was interpreted that the U suture may be more effective than bolster dressing application in controlling the scar tissue in the supratip region. The fact that there is no significant difference between simple suture application (Group 1) and bolster dressing (Group 3) shows that both treatments are similarly effective in scar tissue control.

The ratio of scar thickness to skin thickness indicates the therapeutic effectiveness of the applied surgical techniques. The fact that the ratio of scar thickness to skin thickness was significantly higher in the control group compared with the other groups was another finding showing the positive effects of treatments applied to the supratip area on scar tissue control. No difference between U suture and bolster dressing in terms of scar thickness was found in the ratio of scar thickness to skin thickness. Their therapeutic efficacy was considered to be similar because of similar results in terms of the thickness ratio.

In the simple suture technique, the nasal soft tissue envelope in the supratip region is fixed to the dorsal septum, an adjacent structure. In contrast, in the U suture technique, it is fixed to the columella, a more distant structure. This distant fixation allows the tension on the suture to be adjusted to the extent desired and ensures better adaptation of the soft tissue envelope to the cartilage–bone framework. Hoehne et al. [8] also reported that the U suture is more advantageous in providing suture tension than the simple suture.

Zholtikov et al. [5] developed a percutaneous suture technique (skin contour suture) to decrease dead space, and they demonstrated radiologically that it reduces fibrosis under the skin. These sutures can also be considered an external modification of traditional suture techniques used in the supratip region. The most likely complication due to these suture techniques is a decrease in the perfusion of the skin. No macroscopic or histological perfusion problems were found in any of the subjects. This shows that these suture techniques can be used safely when subcutaneous soft tissues are not trimmed.

Despite its strengths, this study had some limitations. First, immunohistochemical evaluation for fibrosis was not conducted. Future studies should include immunohistochemical evaluation for fibrosis. Second, the follow-up period was relatively short. Since most of the experimental studies on rhinoplasty in rabbits in the literature have a follow-up period of 12 weeks, and the completion period of the study was planned to be 12 weeks [14–17]. It would be helpful to support the findings of our study with data obtained due to scar maturation during longer follow-ups (>12 weeks). Since this study was experimental, the results were evaluated only from a histological aspect. We believe that the results obtained in our research will be guided in terms of clinical applications. Comparative clinical studies on the subject are still needed.

## Conclusion

This study is the most comprehensive experimental model comparing the techniques described in the literature in preventing supratip deformity. The results of this study show that suture techniques or bolster dressings applied in the supratip region have similar effects, and obliteration of the dead space with these techniques has a positive outcome in terms of limiting scar formation.
